# Supplementary material for: Influenza A virus NS1 effector domain is required for PA-X-mediated host shutoff in infected cells
Source: J Virol. 2024 Apr 17;98(5):e01901-23. doi: 10.1128/jvi.01901-23 (PMC11092343; doi:10.1128/jvi.01901-23)
Supplement: Figure S1 — Direct RNA sequencing of nuclear and cytoplasmic poly(A) RNAs using nanopore technology. [file jvi.01901-23-s0001.pdf]

A

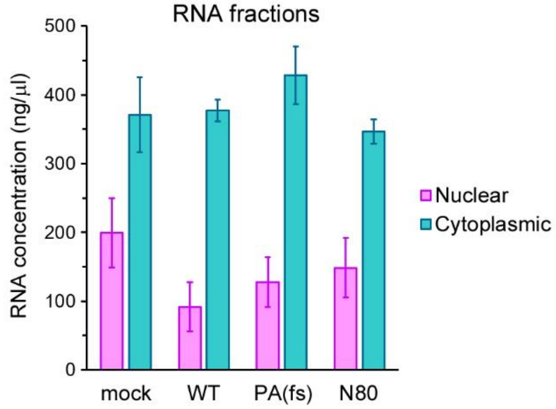

B

Nanopore reads summary

RNA fraction	Nuclear				Cytoplasmic	
PR8 infection	-	WT	PA(fs)	N80	-	WT
Total Reads	1,429,035	1,482,701	1,191,840	1,144,140	1,830,972	1,658,672
Passed reads > 207 nt	1,061,136	1,047,691	919,944	886,248	1,499,990	1,432,026
IAV reads > 207 nt	-	365,314	297,768	128,613	-	327,093
IAV reads with polyA > 207 nt	-	306,790	250,996	103,554	-	287,577
IAV reads poly(A) average length	-	76	95	112	-	78
H.sap reads > 207 nt	538,178	221,834	385,059	636,446	1,364,360	555,872
H.sap reads with polyA > 207 nt	402,043	171,899	298,476	477,389	1,066,205	455,451
H.sap reads poly(A) average length	112	86	93	122	84	68
S.cer ENO2 reads > 207 nt	511,756	447,233	211,632	112,430	121,591	533,553
S.cer ENO2 reads with polyA > 207 nt	380,300	343,378	161,871	83,536	91,873	438,763
S.cer ENO2 poly(A) average length	39	39	39	39	40	40

C

Nuc.

AMOTL2
ARRDC3
CITED2
DUSP6
PCF11
PLEC
RNA5-8SN1
RNA5-8SN2
RNA5-8SN3
RNA5-8SP10
SGK1
SNORD3A
SNORD3B-2
TBX3

Nuc. & Cyt.

BEX2
CCN2
FOS
GADD45A
GADD45B
GEM
HBEGF
JUNB
lnc-IZUMO2-2
lnc-KCNE1B-157
RMND5A
RN7SL1
RN7SL2
RN7SL3
RND3
RNU6-8
RPL26P36
SNHG25
TNFRSF10D
ZFP36L1

Cyt.

ANXA2R-OT1
DDIT3
DNAJB9
H3P36
IER2
JUN
lnc_RNA_ENSG00000290908
lnc-CCNB1IP1-1
MEX3C
MIRLET7A1HG
NACA4P
NIPA1
NUPR1
PMAIP1
PPP1R2
RPL17-C18orf32
RPL17P18
RPL21P19
RPL21P80
RPL26P23
RPL31P55
RPL31P63
RPL35P1
RPL36AP49
RPS11P
RPS11P7
RPS17P5
RPS19P5
RPS20P35
RPS27AP2
RPS2P17
RPS2P31
RPS2P48
SNAI1
SOWAHC

S1 Fig. Direct RNA sequencing of nuclear and cytoplasmic poly(A) RNAs using nanopore technology. (A) Total RNA concentrations in nuclear and cytoplasmic fractions obtained from cell infected with the indicated recombinant mutant viruses or mock infected. Mean values from 6 independent replicates are plotted. Error bars represent standard deviations. (B) Summary of nanopore reads analyses obtained from poly(A) RNAs isolated from the cells infected with the indicated recombinant mutant viruses or mock infected. In each sample, yeast ENO2 spike in control RNA with 30 nt long poly(A) tail was added for comparison. *H.sap* = *Homo sapiens*; *S.cer* = *Saccharomyces cerevisiae.* (C) Human poly(A) transcripts identified in samples from WT PR8 virus-infected cells and absent in mock-infected cell samples. Nuc. = detected only in nuclear RNA sample (red); Nuc. & Cyt. = detected in both nuclear and cytoplasmic samples (orange); Cyt. = detected only in cytoplasmic sample (green).
